# Molecular Engineering of Nicotinamide Riboside Kinase and Process Optimization for Efficient Nicotinamide Mononucleotide Production

**DOI:** 10.3390/foods15111838

**Published:** 2026-05-22

**Authors:** Dai Ma, Rui Liu, Tong Bao, Jingwen Yang, Hongbin Zhang, Xueqin Hu

**Affiliations:** School of Food and Biological Engineering, Hefei University of Technology, Hefei 230000, China; 2023171415@mail.hfut.edu.cn (D.M.);

**Keywords:** nicotinamide mononucleotide synthesis, nicotinamide riboside kinase, molecular engineering, ATP regeneration system, dual-enzyme coupling

## Abstract

Nicotinamide mononucleotide (NMN) plays vital physiological roles as a vitamin B derivative, with nicotinamide riboside kinase (NRK) serving as a key enzyme for its efficient and environmentally friendly synthesis. In this study, semi-rational design was employed to modify the *Hi*-NRK enzyme at the molecular level, leading to the identification of a critical mutant, *Hi*-NRK^G8S^. This variant exhibited a twofold increase in enzymatic activity and significantly enhanced thermal stability, extending its half-life at 40 °C from 4 to 8 h. By optimizing reaction conditions, NMN yield reached 94.17% at a nicotinamide riboside (NR) substrate concentration of 50 g/L. Further addition of adenylate kinase (ADK) to facilitate ATP recycling increased the yield to 97.24% at 75 g/L NR. This study establishes a foundation for industrial-scale, efficient, and green NMN production.

## 1. Introduction

β-Nicotinamide mononucleotide (β-NMN) is a direct precursor of nicotinamide adenine dinucleotide (NAD^+^), which is involved in essential cellular processes such as metabolism, aging, apoptosis, DNA repair, and gene regulation [1,2]. NAD^+^ levels decline with age, making β-NMN a promising supplement for restoring NAD^+^ and a key focus of anti-aging research [3]. Beyond anti-aging, β-NMN has demonstrated pharmacological effects in various diseases, including cardiovascular and neurological disorders, Alzheimer’s disease, type 2 diabetes, and obesity associated with diet and aging [4,5]. Additionally, β-NMN serves as a crucial intermediate in NAD^+^ biotransformation and enzymatic synthesis and can be converted into NADP^+^ [6]. The global market for NMN has experienced remarkable growth in recent years, driven by increasing consumer awareness of health and longevity. Market analyses indicate that the NMN supplement industry is projected to reach billions of dollars in the coming decade, with applications extending from dietary supplements to functional foods and pharmaceutical formulations [7]. This escalating demand necessitates the development of cost-effective and scalable production methods.

NMN production methods primarily fall into two categories: chemical synthesis and biosynthesis. Chemical approaches typically involve Vorbrüggen glycosylation, phosphorylation, and deamination steps [8]. Although industrialized, chemical synthesis suffers from low efficiency, environmental concerns, and frequent formation of the biologically inactive α-NMN isomer [9]. Specifically, the Vorbrüggen glycosylation reaction requires harsh conditions including the use of toxic heavy metal salts as catalysts and expensive protecting groups, which not only increases production costs but also generates substantial hazardous waste. Furthermore, the stereoselectivity of chemical glycosylation remains suboptimal, typically producing mixtures of α- and β-anomers that require tedious chromatographic separation, significantly reducing overall process efficiency and atom economy [8,9].

**Figure 1:**
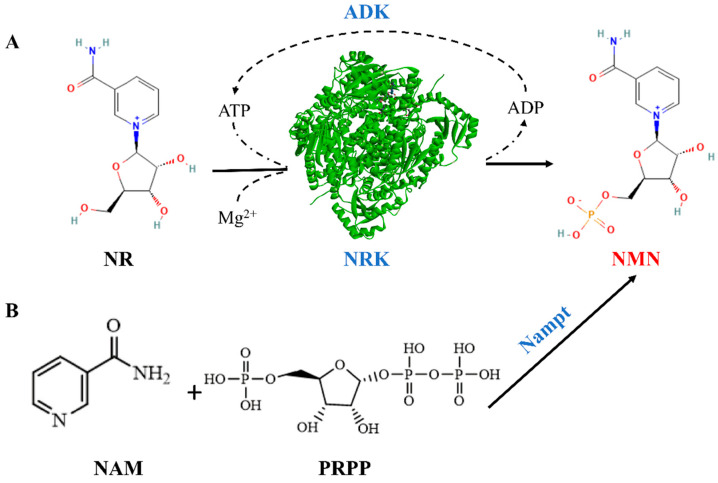
Schematic illustration of the biocatalytic strategy and workflow. (**A**) Enzymatic phosphorylation of NR to NMN coupled with ATP regeneration. (**B**) Biosynthesis of NMN using nicotinamide as a substrate.

In contrast, biocatalytic approaches have gained attention for NMN production due to their non-toxic nature, high efficiency, product selectivity, and mild reaction conditions. Enzyme-catalyzed reactions inherently exhibit exquisite regio- and stereoselectivity, ensuring exclusive formation of the biologically active β-NMN isomer without the need for subsequent separation steps. Moreover, the aqueous reaction medium and biodegradable catalysts align with the principles of green chemistry, making biocatalysis an environmentally sustainable alternative to traditional chemical manufacturing [10]. Two main biosynthetic pathways exist [11] (Figure 1): one catalyzed by nicotinamide phosphoribosyltransferase (Nampt), which converts phosphoribosyl pyrophosphate (PRPP) and nicotinamide (NAM) into NMN [12]; the other utilizes nicotinamide riboside (NR) and ATP as substrates under nicotinamide riboside kinase (NRK) catalysis to produce NMN [13]. The latter pathway is more direct, avoiding intermediates, and offers higher conversion efficiency and lower costs [14], making it more suitable for industrial applications.

Despite the potential of NRK-mediated NMN synthesis, several challenges persist in translating this biocatalytic system to industrial practice. Wild-type NRKs typically exhibit limited catalytic efficiency and insufficient thermostability, restricting their operational lifespan under process conditions. The requirement for stoichiometric amounts of ATP as the phosphate donor further escalates production costs, necessitating the integration of efficient cofactor regeneration systems. Furthermore, substrate inhibition at elevated NR concentrations commonly encountered in industrial settings compromises overall process productivity. Addressing these limitations through rational enzyme engineering and reaction engineering strategies has become a key research point to improve the catalytic activity of NRKs [15].

In this study, we aimed to obtain a high-activity nicotinamide riboside kinase through combinatorial semi-rational design. By leveraging molecular docking and molecular dynamics simulations to identify hotspot residues for mutagenesis, combined with saturation mutagenesis and combinatorial library construction, this strategy enables efficient navigation of sequence space with reduced experimental burden. This design significantly improves the efficiency and stability of β-NMN synthesis. Detailed analysis of the interactions between the variants and the substrates provided valuable insights into the enzyme-catalyzed process. Adenylate kinase (ADK) was introduced to build the ATP-cycling system to ultimately realize the efficient synthesis of β-NMN. This work not only provides a robust biocatalyst for β-NMN biosynthesis but also offers an alternative route for the sustainable and economic production of β-NMN. Our work lays the foundation for large-scale fermentation and low-cost production in the industrial production of β-NMN, thus further increasing the commercial application of NMN in food and pharmaceutical industries.

## 2. Materials and Methods

### 2.1. Strains, Plasmids, and Reagents

Genes encoding *Hi*-NRK (GenBank: KPH72847.1) and ADK (GenBank: GQ249079) were codon-optimized for *Escherichia coli* and synthesized commercially [16]. These genes were cloned into the pET28a vector at the BamHI and XhoI sites to generate the pET28a/*Hi*-NRK and pET28a/ADK plasmids, which were confirmed by sequencing. *E. coli* DH5α was used for cloning and plasmid maintenance, while BL21 (DE3) was employed for protein expression. Enzymes for molecular biology and kits for PCR and plasmid preparation were purchased from TSINGKE (Nanjing, China). High-purity NR and β-NMN were provided by the Shanghai Macklin Biochemical Technology Co., Ltd. (Shanghai, China). All other reagents were of analytical grade. LB medium was prepared using tryptone, yeast extract, and NaCl. Other chemicals and standard samples were purchased from Aladdin (Shanghai, China).

### 2.2. Mutant Design and Construction

To optimize electrostatic interactions between NRK and the ATP phosphate group, site-directed mutagenesis was performed within the ATP-binding loop of *Hi*-NRK. The target residue was selected based on molecular docking and sequence alignment analyses. Alanine scanning of the ATP-binding loop residues was conducted to systematically assess the contribution of each position to enzyme function, identifying promising targets for saturation mutagenesis. PCR amplification was performed using designed primers (Table S1), followed by DpnI digestion and transformation into *E. coli* BL21. PCR amplification was performed following standard protocols (Tables S2 and S3). Positive clones were verified by sequencing.

### 2.3. Protein Expression and Purification

Recombinant plasmids were transformed into *E. coli* BL21(DE3). Cultures were grown in LB medium containing kanamycin at 37 °C and 220 rpm until the OD_600_ of 1.8–2.0 was reached, then induced with IPTG (final concentration of 0.0625 mg/mL) at 25 °C for 12 h. Cells were harvested and lysed by sonication, and the supernatants were purified using Ni-NTA affinity chromatography. Purity was assessed by SDS-PAGE, and protein concentration was determined by the Bradford assay [17].

### 2.4. Enzyme Activity Assay

Determination of *Hi*-NRK activity. The enzymatic reaction was initiated by adding 100 μL of appropriately diluted *Hi*-NRK enzyme solution (0.2 mg/mL) to 400 μL of substrate mixture containing 100 mmol/L NR, 100 mmol/L ATP, and 20 mmol/L MgCl_2_, all prepared in 20 mmol/L Tris-HCl buffer (pH 7.5). The reaction was carried out at 40 °C for 1 h and subsequently terminated by boiling. After centrifugation, the supernatants were subjected to HPLC analysis. NR consumption and NMN production were quantified to evaluate the conversion efficiency and yield [18,19]. One unit (U) of *Hi*-NRK activity was defined as the amount of enzyme required to catalyze the formation of 1 μmol of NMN per minute under the standard assay conditions.

Determination of ADK activity. The reaction mixture, consisting of 100 mmol/L ADP and 20 mmol/L MgCl_2_ in 20 mmol/L Tris-HCl buffer (pH 7.5), was preheated to 40 °C. The enzymatic reaction was initiated by adding 100 μL of crude ADK enzyme solution (0.2 mg/mL) to 400 μL of the preheated substrate mixture, followed by rapid mixing and incubation at 40 °C for 10 min. The reaction was terminated by boiling, and the ATP yield derived from ADP was analyzed by HPLC. One unit of ADK enzyme activity (U) was defined as the amount of enzyme required to produce 1 µmol of ATP per minute under the standard assay conditions described above.

The chromatographic column was TSKgel Amide-80 (4.6 mm × 25 cm, Tosoh Bioscience, Tokyo, Japan), the mobile phase was acetonitrile/water (65:35, *v*/*v*) with a flow rate of 1.0 mL/min. The detection wavelength was set at 260 nm.

### 2.5. Kinetic and Enzymatic Characterization

Enzyme kinetics were determined at 40 °C in 20 mmol/L Tris-HCl buffer (pH 7.5). For the determination of the apparent *K_M_* for NR, reactions were initiated by adding appropriately diluted *Hi*-NRK to a mixture containing 2.0 mmol/L ATP and varying concentrations of NR (0–2.0 mmol/L). For the determination of the apparent *K_M_* for ATP, NR was fixed at 2.0 mmol/L and ATP was varied (0–2.0 mmol/L) [20]. Reactions were quenched at 5 min, and initial rates were measured in triplicate. The *K_M_* and *K_cat_* of the enzyme were calculated using Lineweaver-Burk plots. One unit of enzyme activity was defined as the amount catalyzing the formation of 1 μmol of NMN or ATP per minute. Optimal pH and temperature were determined using appropriate buffers and temperature ranges [21]. Thermal stability was assessed by incubating the enzymes at various temperatures and measuring residual activity over time [22].

### 2.6. Molecular Docking and Molecular Dynamics (MD) Simulations

The 3D structure of *Hi*-NRK was modeled using AlphaFold3 (https://alphafoldserver.com/), selecting the highest-scoring model [23]. Ligand structures were obtained from the Protein Data Bank (PDB). Molecular docking was performed with AutoDock Vina (version 1.1.2), and interactions were visualized using PyMOL (version 2.5.0) [24]. Multiple sequence alignments were conducted using UniProt (https://www.uniprot.org) and ESPript 3.2 (https://espript.ibcp.fr/ESPript/cgi-bin/ESPript.cgi, accessed on 13 April 2026) [25].

MD simulations were performed using the GROMACS 2025.5 software package. The docked enzyme-substrate complexes were solvated in a cubic box of TIP3P water molecules, extending at least 1.2 nm from the protein surface, and 0.9% NaCl (*w*/*v*) was added to each system for charge neutralization. Energy minimization was conducted using the steepest descent algorithm, followed by a simulated annealing protocol to relieve unfavorable contacts and achieve a stable starting configuration, under the CHARMM36 all-atom force field with CMAP corrections for backbone dihedrals. The ATP cofactor and Mg^2+^ ions were parameterized using the CHARMM36 nucleotide and standard ion parameters, respectively. The system was subsequently equilibrated using NVT (constant particle number, volume, and temperature) and NPT (constant particle number, pressure, and temperature) ensembles. A production MD simulation was then run for 100 ns at 313 K (40 °C) (to simulate thermal stress) and 1 atm, with a 2 fs time step. Trajectory frames were saved every 100 ps. System stability was evaluated by monitoring the RMSD of Cα atoms, total system energy, and density fluctuations over the simulation time, confirming convergence prior to analysis. The trajectories were processed and analyzed using GROMACS 2025.5 tools to calculate the root-mean-square deviation (RMSD), root-mean-square fluctuation (RMSF), binding free energy, enzyme-substrate distance variations, and dynamic cross-correlation matrix (DCCM) between protein and ligand [26]. Trajectory data and analysis scripts have been deposited in Zenodo (DOI: 10.5281/zenodo.20263633).

### 2.7. ATP Recycling System Construction

Enzymatic reactions were carried out in a 10 mL total volume containing 300 mM nicotinamide riboside (NR), with ATP and MgCl_2_ maintained at a molar ratio of 5:1, in 20 mmol/L Tris-HCl buffer (pH 7.5). *Hi*-NRK and ADK were added at final concentrations of 30 U/mL and 10 U/mL, respectively. The reaction mixture was incubated at 40 °C and pH 7.5 for 3 h with continuous agitation at 200 rpm.

### 2.8. Data Analysis

The data presented in the manuscript were calculated and illustrated using Origin 2024. Each experimental datapoint is the average of three parallel experiments and the experimental standard deviation is indicated by the error bars. *p* < 0.05 was considered to be statistically significant.

## 3. Results

### 3.1. Structural Modeling and Analysis

Using available NRK and NadR crystal structures as templates, a *Hi*-NRK model was generated [27,28]. The stereochemical quality of the generated *Hi*-NRK model was assessed using Ramachandran plot analysis. The results revealed that 90.01% of residues were located in the most favored regions, 6.44% in the additional allowed regions, and 2.55% in the generously allowed regions, with no residues falling within disallowed regions (Figure S1). These statistics indicate that the backbone geometry of the model is reliable and satisfies the criteria for high-quality homology models, providing a trustworthy foundation for subsequent molecular docking and dynamics studies.

**Figure 2:**
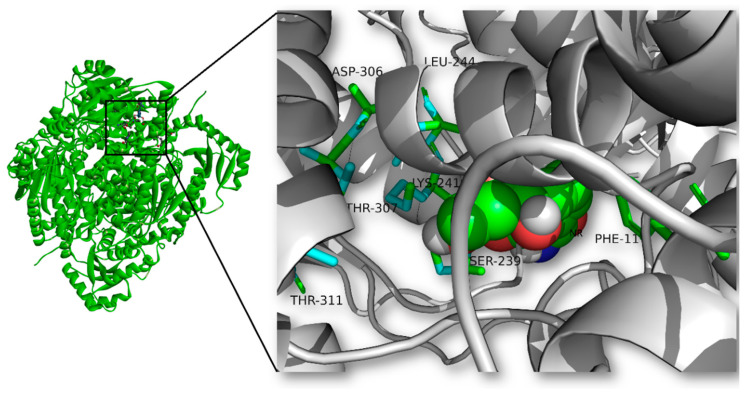
Three-dimensional architecture of *Hi*-NRK^WT^ highlighting the substrate-binding pocket. The global structure is presented in cartoon mode, with an expanded view depicting the key residues (Phe11, Ser239, Lys241, Leu244, Asp306, Thr307, and Thr311) in stick representation.

The three-dimensional architecture of *Hi*-NRK reveals a typical Rossmann fold characteristic of the kinase superfamily [28], comprising a central β-sheet flanked by α-helices (Figure 2). The substrate-binding pocket is formed by residues from both the N-terminal and C-terminal domains, creating a deep cleft that accommodates the nicotinamide riboside moiety. The substrate (NR) was docked into the predicted substrate-binding pocket of *Hi*-NRK using AutoDock Vina. Key catalytic residues Lys241 and Asp306 were identified. Lys241 is positioned to stabilize the transition state through electrostatic interactions with the phosphate moiety during phosphoryl transfer, while Asp306 likely functions as a catalytic base facilitating proton abstraction from the ribosyl hydroxyl group. Docking studies revealed that NR’s hydroxyl group interacts with Gly240 and Asp308, while Arg12 functions as a general base (Figure 2). These interactions collectively establish a precise geometric arrangement that orients the substrate for in-line phosphoryl transfer, consistent with the canonical kinase reaction mechanism involving a nucleophilic attack on the γ-phosphate of ATP [29].

### 3.2. Protein Engineering

NRKs require precise ATP positioning for efficient phosphate transfer to the substrate [30]. Alanine scanning of residues near the catalytic triad reduced enzyme activity, highlighting their critical roles. Meanwhile, molecular docking revealed that Gly8 and Phe266 are located proximal to the ATP-binding loop region and spatially adjacent to the γ-phosphate of ATP in the docked complex. To reduce steric hindrance and introduce favorable hydrogen bonding interactions with the ATP phosphate tail, thereby stabilizing the transition state and improving catalytic efficiency, we performed saturation mutagenesis at Gly8 and Phe266. We identified the G8S and F266W mutants, both demonstrating significantly improved activity.

**Figure 3:**
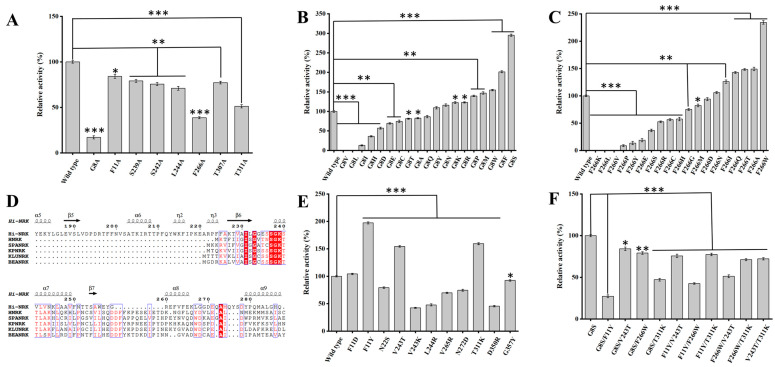
Relative activities of NRK variants. (**A**) Relative activities of NRK variants obtained by alanine scanning. (**B**) Saturation mutagenesis results at Gly8 and (**C**) Phe266. (**D**) Sequence alignment. (**E**) NRK variants with site-directed mutations. (**F**) Two-point combination mutation. Error bars are SD (standard deviation), *n* = 3; * *p*-value < 0.05, ** *p*-value < 0.01, *** *p*-value < 0.001.

Sequence alignment with other NRKs guided site-directed mutagenesis (Figure S2). Notably, the highly conserved GxxxSGKT sequence in NRK, known as the P-loop or Walker A motif, is a key structural element essential for NR phosphorylation [31]. To preserve its functional integrity, no mutations were introduced in this region. Mutations targeting residues near the NR and ATP binding sites were designed to reduce steric hindrance and increase hydrophilicity. Variants F11Y, V243T, and T311K exhibited more than a 1.5-fold increase in activity, with F11Y showing the greatest enhancement. The subsequent iterative combination mutation based on these favorable mutants was performed, but the effect is not ideal, which may be due to an epistatic effect [32] (Figure 3).

### 3.3. Structural Analysis of Hi-NRK^WT^ and Hi-NRK^G8S^

**Figure 4:**
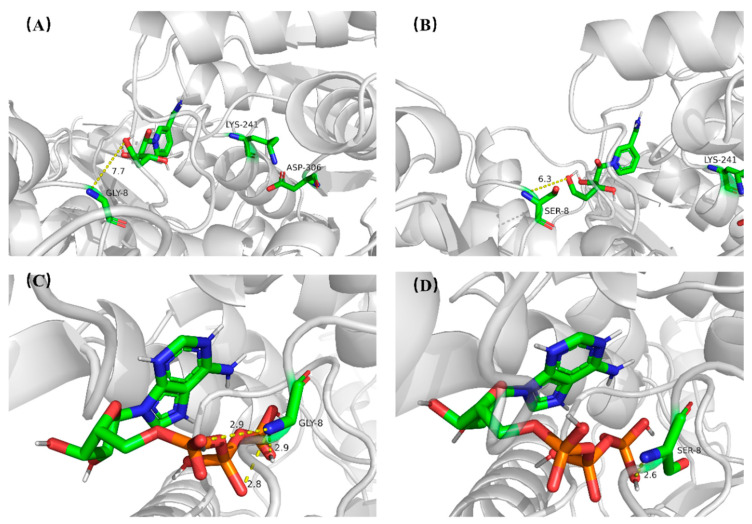
In silico analysis of ligand binding modes in *Hi*-NRK^WT^ and *Hi*-NRK^G8S^. (**A**) The interaction of Gly8 with NR in *Hi*-NRK^WT^. (**B**) The interaction of Ser8 with NR in *Hi*-NRK^G8S^. (**C**) The interaction of Gly8 with ATP in *Hi*-NRK^WT^. (**D**) The interaction of Ser8 with ATP in *Hi*-NRK^G8S^. The protein backbone is illustrated in cartoon representation, while ligands and critical side chains are displayed as sticks.

Although the distance between the amino group of Gly8/Ser8 and the hydroxyl group of the substrate NR exceeds 6 Å (Figure 4A,B), the G8S substitution has a significant effect, suggesting that there may be no direct interaction between NR and Gly8/Ser8. Docking analysis revealed differences in how Ser8 and Gly8 interact with the co-substrate ATP in *Hi*-NRK^WT^ and *Hi*-NRK^G8S^ (Figure 4C,D). Specifically, the main-chain amino and carboxyl groups of residue 8 are structurally poised to modulate ATP hydrolysis. Notably, the hydrogen-bonding arrangements involving residue 8 and the nucleotide co-substrate diverge markedly between *Hi*-NRK^WT^ and *Hi*-NRK^G8S^. In *Hi*-NRK^WT^, these non-covalent contacts are distributed across various phosphate moieties of ATP. Conversely, the G8S substitution constrains Ser8 to engage predominantly with the γ-phosphate, a configuration that could promote phosphoanhydride bond cleavage and ADP release. This proposed interaction pattern is consistent with the reported catalytic mechanism of NRK [33], though further experimental validation is required to confirm its functional significance. However, limited structural data on NRKs may affect the accuracy of the modeling.

**Figure 5:**
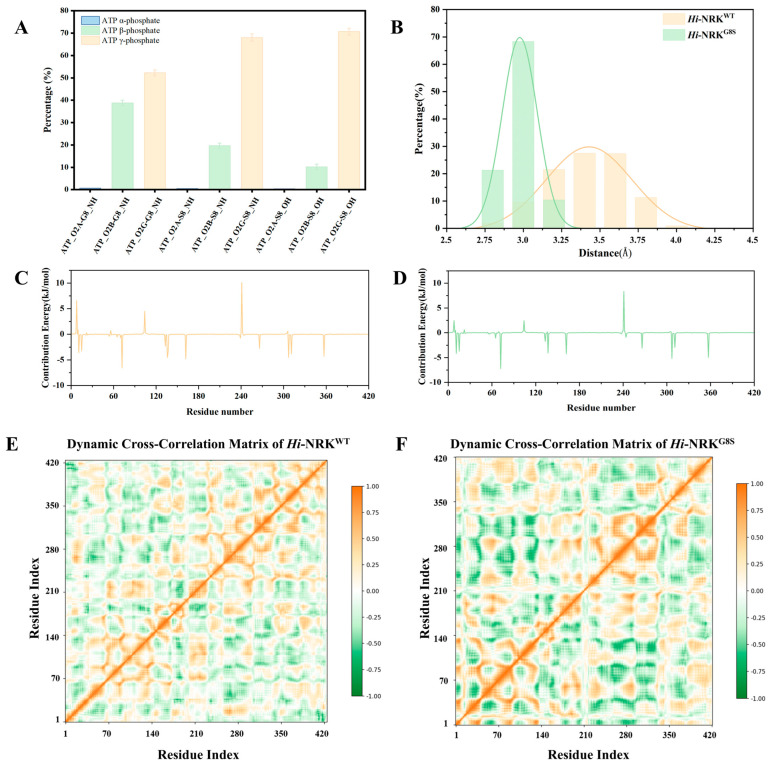
Molecular dynamics simulation analyses for *Hi*-NRK^WT^ and *Hi*-NRK^G8S^. (**A**) Hydrogen bond percentage of the residue 8. (**B**) Frequency distribution histogram of the distance between the enzyme and the substrate in the complex. The per-residue energy contributions based on MM-PBSA-binding affinity calculations (**C**) *Hi*-NRK^WT^ and (**D**) *Hi*-NRK^G8S^. Dynamic cross-correlation matrices (DCCM) for *Hi*-NRK^WT^ (**E**) and *Hi*-NRK^G8S^ (**F**). Error bars are SD, *n* = 3.

To quantitatively characterize these differential ATP-binding modes, 100 ns molecular dynamics (MD) simulations were performed at 313 K (40 °C) for both *Hi*-NRK^WT^ and *Hi*-NRK^G8S^. Hydrogen bond occupancy analysis revealed striking differences in how residue 8 engages with ATP phosphate oxygens (Figure 5A). In *Hi*-NRK^WT^ (Gly8), the backbone NH interacts with all three phosphate oxygens but predominantly with the β- and γ-phosphates (ATP_O2B: 38.78%, ATP_O2G: 52.23%); the α-phosphate interaction is negligible (ATP_O2A: 0.71%). This gradient reflects the inherent tendency of the P-loop to position the triphosphate chain for catalysis; however, the flexible glycine-rich loop permits substantial β-phosphate engagement, indicating a relatively flat binding geometry. In *Hi*-NRK^G8S^ (Ser8), the hydrogen-bonding landscape is dramatically restructured. The backbone NH shows enhanced γ-phosphate engagement (68.03% at ATP_O2G, compared to 52.23% in WT), accompanied by reduced interactions with distal phosphates (ATP_O2A: 0.46%, ATP_O2B: 19.64%). More critically, the serine side-chain hydroxyl introduces a new, highly selective hydrogen bond donor that almost exclusively targets the γ-phosphate oxygen ATP_O2G (70.7% occupancy, average donor-acceptor distance 2.82 ± 0.15 Å). The side-chain OH shows minimal interaction with α- and β-phosphates (0.33% and 10.13%, respectively), demonstrating that the G8S mutation does not merely add another hydrogen bond but reprograms the P-loop from a distributed, flexible anchor to a precision-guided γ-phosphate clamp.

Distance distribution analysis corroborates this geometric precision (Figure 5B). *Hi*-NRK^WT^ exhibits broader distance fluctuations between the substrate and catalytic residues, whereas *Hi*-NRK^G8S^ displays a sharp Gaussian peak centered at a catalytically productive distance, consistent with a more rigid active-site pocket. A more rigid active-site pocket environment is conducive to precise substrate binding and positioning, which further explains the mutant’s ability to retain high activity from the perspective of substrate binding. The per-residue binding free energy decomposition based on MM-PBSA calculations provides energetic insights into this stabilization mechanism (Figure 5C,D). In *Hi*-NRK^G8S^, the total binding free energy decreased to −32.49 kJ/mol, significantly lower than that of the wild-type (−27.17 kJ/mol), indicating an approximately 19.6% enhancement in binding affinity. This stabilization primarily stems from improved electrostatic interactions in the P-loop region (residues 235–242), where the Ser8 hydroxyl reorients the backbone to optimize Lys241 coordination with the γ-phosphate [34,35]. The tightened phosphate positioning enhances electrostatic pre-organization of the active site, reducing the reorganization energy penalty upon ATP binding [36].

This serine latch mechanism where a P-loop side-chain hydroxyl selectively clamps the γ-phosphate is conserved across related kinases. In thymidylate kinase, a P-loop serine hydrogen-bonds with the γ-phosphate to facilitate in-line attack [37]. Similarly, human deoxycytidine kinase employs P-loop serine or threonine residues to stabilize the transition state [38]. The G8S mutation in *Hi*-NRK introduces an analogous mechanism, where Ser8_OH pre-orients the γ-phosphate for nucleophilic attack by the ribosyl hydroxyl of NR. Dynamic cross-correlation matrix (DCCM) analysis (Figure 5E,F) further reveals enhanced correlation between the P-loop (site 241) and the substrate-binding groove (sites 270–310) in *Hi*-NRK^G8S^ [28,39], suggesting tighter coupling between ATP binding and phosphate guidance. The Ser8_OH—ATP_O2G hydrogen bond thus functions as a conformational anchor that restricts P-loop flexibility while maintaining catalytically productive geometry, consistent with the established role of Walker A motif pre-organization in minimizing the entropic cost of nucleotide binding [34,35].

### 3.4. Characterization of the Enzymatic Properties of Hi-NRK^WT^ and Hi-NRK^G8S^

**Figure 6:**
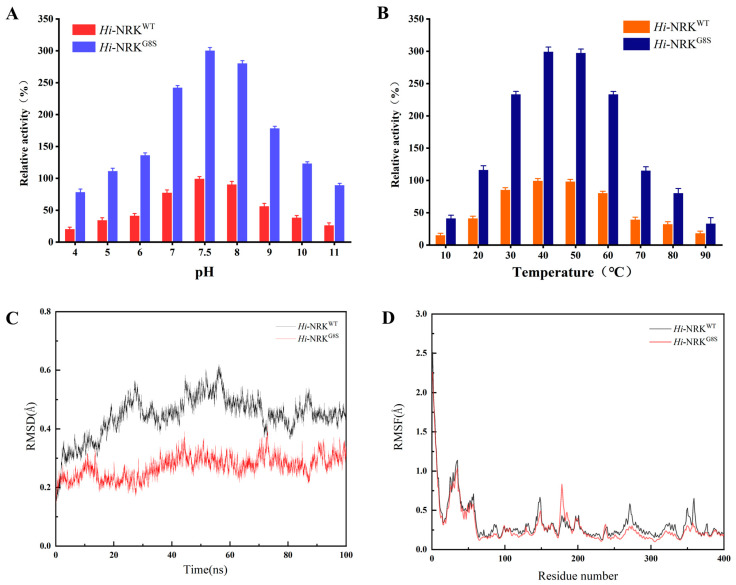
Effects of pH (**A**) and temperature (**B**) on *Hi*-NRK activity and stability. Molecular dynamics simulation analyses for *Hi*-NRK^WT^ and *Hi*-NRK^G8S^. (**C**) Backbone Cα atom root-mean-square deviation (RMSD) over time. (**D**) Per-residue root-mean-square fluctuation (RMSF). Error bars are SD, *n* = 3.

The enzyme was purified to homogeneity by Ni-NTA affinity chromatography, and the purity was assessed by SDS-PAGE (Figure S3). The activities of *Hi*-NRK^WT^ and its variant *Hi*-NRK^G8S^ were measured across different pH values. The optimal pH for *Hi*-NRK^G8S^ was determined to be 7.5 (Tris-HCl buffer), showing no significant difference compared to *Hi*-NRK^WT^ (Figure 6A). The temperature dependence of *Hi*-NRK catalytic efficiency was further assessed across 10–90 °C (Figure 6B). The results indicated that both *Hi*-NRK^WT^ and *Hi*-NRK^G8S^ displayed maximal activity near 40 °C. The broad temperature profile, with substantial activity maintained from 30 °C to 50 °C, offers operational flexibility for process design and suggests that the enzyme retains functional conformation across a moderately wide thermal range. Comparative thermostability profiling at this temperature revealed marked differences among variants: the half-life of *Hi*-NRK^WT^ was only 4 h, whereas *Hi*-NRK^G8S^ and *Hi*-NRK^F11Y^ required 8 h and 13 h to reach equivalent inactivation (Figure S4). These observations implied that advantageous mutations concurrently bolstered catalytic performance and conformational resilience under thermal stress.

To elucidate the structural underpinnings of these phenomena, atomistic MD simulations were conducted at 313 K (40 °C) for *Hi*-NRK^WT^ and *Hi*-NRK^G8S^. Time-averaged backbone RMSD (Figure 6C) over the terminal 10 ns trajectory segments indicated superior structural rigidity in *Hi*-NRK^G8S^ (0.26 Å) relative to *Hi*-NRK^WT^ (0.41 Å), signifying attenuated backbone mobility in the mutant. This global rigidification is mechanistically linked to the ATP_O2G-Ser8_OH hydrogen bond, which acts as a conformational anchor restricting P-loop mobility while maintaining the loop in a catalytically productive geometry. RMSF (Figure 6D) analysis revealed that *Hi*-NRK^G8S^ exhibits reduced fluctuation amplitudes across most of the polypeptide chain, consistent with the RMSD trend. Notably, regions distant from the active site, including the C-terminus, showed enhanced rigidity in the mutant, suggesting that the P-loop stabilization propagates cooperatively to distal structural elements.

The thermostability enhancement conferred by the G8S mutation stems from entropic restriction of P-loop flexibility. Glycine, with its minimal side chain, confers high backbone flexibility to the P-loop, a feature evolutionarily conserved to accommodate diverse nucleotide conformations [40]. However, this flexibility renders the loop susceptible to thermal denaturation, which frequently initiates at dynamic regions [41]. The serine substitution introduces side-chain hydrogen bonding that constrains the P-loop conformation, reducing its conformational entropy and raising the free energy barrier for unfolding. This mechanism is directly supported by the hydrogen bond occupancy data, the persistent ATP_O2G-Ser8_OH interaction (70.7% occupancy) restricts the P-loop to a catalytically competent conformation, whereas Gly8 in *Hi*-NRK^WT^ permits multiple suboptimal states. The extended half-life of *Hi*-NRK^G8S^ at 40 °C reflects this enhanced conformational resilience.

### 3.5. Kinetic Analysis of Hi-NRK and Its Mutants

Compared to previously reported NRKs from various organisms (Table 1), the *Hi*-NRK^G8S^ exhibits competitive kinetic parameters, with the catalytic efficiency for NR (43.31 s^−1^·mM^−1^) surpassing that of most characterized homologs [13] (Figure S5). The analysis revealed that the G8S mutant’s *K_M_* for ATP decreased from 126 μM to 42 μM, while the *K_M_* for NR decreased from 85 μM to 65 μM. Additionally, the G8S mutant exhibited increased *k_cat_* values for both the primary substrate and co-substrate, resulting in a fivefold increase in catalytic efficiency (*k_cat_*/*K_M_*) for ATP and a twofold increase for NR. The kinetic data indicated that the *Hi*-NRK^G8S^ mutant has a significantly enhanced affinity for ATP, nearly three times higher than that of the wild-type enzyme. This improvement substantially enhances the conversion efficiency of NR. In the *Hi*-NRK^G8S^ variant, residue S8 primarily formed hydrogen bonds with the γ-phosphate moiety of ATP based on molecular docking and MD simulations. This specific hydrogen bonding is hypothesized to facilitate the phosphoryl transfer from ATP to NR, consistent with the observed kinetic enhancement.

### 3.6. ATP Recycling System

**Figure 7:**
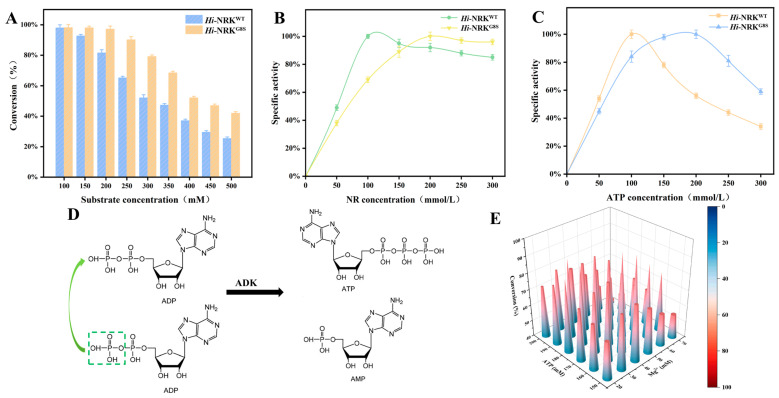
(**A**) Conversion rates of *Hi*-NRK^G8S^ at different substrate concentrations. Enzyme activity at different NR (**B**) and ATP (**C**) concentrations. (**D**) Reaction equation for ATP regeneration. (**E**) Optimization of ATP and Mg^2+^ concentrations in the catalytic reactions of *Hi*-NRK^G8S^ and ADK. Error bars are SD, *n* = 3.

Enzymatic phosphorylation requires a substantial amount of ATP as the phosphate donor. As the concentration of NR increases, this cost rises significantly. Figure 7A shows that when the NR concentration increased to 500 mM, the final conversion rate was only about 45%, possibly due to substrate inhibition at high substrate load. As shown in Figure 7B,C, we detected the changes in enzyme activity in *Hi*-NRK^G8S^ under different NR and ATP concentrations and found that high concentration of ATP was the main reason for the decline of enzyme activity (*Hi*-NRK^G8S^ activity: 3.10 U·mg^−1^ at 200 mM ATP, 1.90 U·mg^−1^ at 300 mM ATP). The observed ATP inhibition likely results from non-productive binding of excess nucleotide at the active site or allosteric regulatory sites, disrupting the precise geometric arrangement required for efficient phosphoryl transfer. This phenomenon is common among nucleotide-dependent enzymes. To overcome this limitation, we subsequently developed an ATP recycling system for NMN biosynthesis to reduce raw material costs and alleviate inhibition caused by high substrate concentrations. This approach also minimizes downstream processing requirements, thereby reducing the time needed for product separation and purification.

In this biosystem, ADK from Sinorhizobium sp. NP1 (GenBank ID: GQ249079), previously reported as an ATP regeneration enzyme, was utilized [42]. Using ATP as the phosphate donor, NR is first phosphorylated to NMN by NRK in the presence of phosphate. Subsequently, ADK regenerates one molecule of ATP from two molecules of ADP, accompanied by the production of one molecule of AMP (Figure 7D). To ensure efficient reaction progress and reduce ATP raw material costs, the initial ATP concentration in the coupled reaction was optimized. The *K_M_* of NR is approximately 1.5 times that of ATP, indicating that to achieve maximum reaction velocity, the amount of NR should be at least 1.5 times that of ATP. As shown in Figure 7E, an initial ATP concentration above 160 mM favors the coupled reaction, with the most economical raw material ratio being NR:ATP = 300 mM:160 mM (with 40 mM Mg^2+^). Under optimal reaction conditions, an NMN concentration of 291.72 mM (97.5 g/L) was obtained, corresponding to a product yield of 97.24%. The collected NMN solution was purified via macroporous adsorption resin chromatography. The resulting eluate was subsequently concentrated and lyophilized to obtain NMN with a purity exceeding 95% (Figures S6 and S7).

Comparison with previously reported ATP recycling systems for NMN synthesis (Table 2) reveals that the combination of engineered *Hi*-NRK^G8S^ with ADK achieves not only the highest reported NMN titer but also exceptional conversion efficiency, surpassing systems employing polyphosphate-dependent kinases or alternative cofactor regeneration strategies [18,19,29,33,43,44,45,46]. The simplicity of the ADK-based recycling system, requiring only ADP as the recycled intermediate without the need for expensive polyphosphate substrates or additional auxiliary enzymes, contributes to its economic attractiveness and operational robustness.

## 4. Discussion

The expanding applications of NMN as a NAD^+^ precursor in nutraceutical and pharmaceutical sectors have intensified efforts to develop efficient biocatalysts for its sustainable production. In this study, we employed a combinatorial semi-rational design strategy to engineer *Hi*-NRK, identifying the G8S mutation as a critical variant that simultaneously enhances catalytic activity and thermal stability. The resulting biocatalyst achieves 94.17% NMN yield at 50 g/L NR, and integration with an ADK-mediated ATP recycling system further elevates the yield to 97.24% at 75 g/L NR with a productivity of 32.50 g/L/h. These performance metrics compare favorably with recently reported NRK variants [13,33], while additionally addressing the persistent thermal instability that constrains industrial deployment of mesophilic NRKs.

The structure-guided approach integrates molecular docking, alanine scanning, and saturation mutagenesis to navigate sequence space surrounding the P-loop region. This methodology proves particularly effective for NRK, where the conserved Walker A motif imposes strict constraints on direct mutagenesis yet harbors proximal residues amenable to optimization [14,47]. The G8S mutation exemplifies how subtle alterations in loop dynamics can profoundly influence catalytic efficiency without disrupting essential structural elements, resonating with independent observations that the G13S mutation in human NRK2 similarly enhances activity through modulation of ATP-binding geometry [14]. This convergence suggests a potentially generalizable principle: P-loop-adjacent glycine-to-serine substitutions can reprogram nucleotide binding dynamics in NRKs, offering a rational target for future engineering across diverse kinase homologs. Nevertheless, this interpretation relies substantially on molecular dynamics simulations without direct structural validation of the ternary complex; definitive confirmation through X-ray crystallography or cryo-electron microscopy is required to validate the proposed mechanism and guide subsequent design efforts, particularly given the limited NRK crystal structures beyond the human ortholog [28,47].

The ADK-coupled ATP recycling system represents a deliberate simplification of cofactor regeneration relative to polyphosphate-dependent alternatives [18,19,29]. While acetate kinase or polyphosphate kinase systems achieve comparable recycling efficiency, they necessitate expensive polyphosphate substrates and additional auxiliary enzymes that complicate process economics [18,19]. The ADK-mediated equilibrium exploits commercially available ADP as the sole recycled intermediate, reducing biocatalyst complexity and achieving 97.24% yield at 97.5 g/L NMN. However, this batch performance at 10 mL scale remains to be benchmarked against continuous-flow whole-cell systems that have demonstrated superior space-time yields through process intensification [14], and the substrate concentrations employed, though exceeding many prior studies, remain modest relative to industrial targets exceeding 200 g/L where substrate inhibition and viscosity effects become pronounced [44]. The absence of scale-up data, immobilization studies, or continuous process evaluation further separates this work from demonstrated industrial feasibility, as batch operations cannot reliably predict performance in stirred-tank or fixed-bed reactors subject to mass transfer limitations and prolonged operational stress [48]. Additionally, reliance on *Escherichia coli* expression with standard Ni-NTA purification incurs significant production costs and raises endotoxin concerns for food-grade applications, suggesting the need for future transition to GRAS-certified hosts or cell-free expression systems [49].

These constraints illuminate productive directions for continued development. Machine learning-guided protein design may circumvent epistatic limitations encountered in iterative mutagenesis, identifying distal mutations that allosterically modulate P-loop dynamics [50]. Metabolic engineering through CRISPR/Cas9-mediated knockout of endogenous degradation pathways (*deoD*, *ushA*, *pncC*, *nadR*) would improve substrate utilization as demonstrated in recent studies [14,51], while process intensification via enzyme immobilization on functionalized carriers or cross-linked enzyme aggregates could enable continuous operation with enhanced stability [48]. Ultimately, de novo biosynthesis from inexpensive carbohydrate feedstocks through multi-enzyme cascades would eliminate dependence on chemically synthesized NR, transforming NMN production into a fully sustainable biomanufacturing process.

## 5. Conclusions

NRKs with potential for industrial applications should exhibit favorable enzymatic properties, particularly high catalytic activity. This study explored the enhancement of nicotinamide riboside kinase activity and stability through molecular modification, with the G8S mutation showing improved substrate binding and phosphate transfer efficiency in vitro. By coupling NRK with ADK, ATP recycling was demonstrated, enabling substantial conversion of elevated NR concentrations within three hours under optimized batch conditions. While these findings suggest a promising direction for NMN biosynthesis, further evaluation of long-term stability, productivity, and process scalability will be necessary to assess industrial feasibility. The developed method provides a foundation for future optimization and may offer insights for the design of cost-effective biocatalytic strategies.

## 6. Patents

Hu X Q, Ma D, Xia B B, Zhang H B, Yang J W. A recombinant *Escherichia coli* producing nicotinamide riboside kinase and its construction method and application. Patent CN119060982B. 2025.

Hu X Q, Ma D, Zhang H B, Yang J W, Gao Y F. A β-nicotinamide mononucleotide production system and a method for synergistic preparation of β-nicotinamide mononucleotide by dual enzymes. Patent CN120025959B. 2025.

## Figures and Tables

**Table 1:** Kinetic Parameters of NRKs.

NRK	*K_M_* (μM)	*k_cat_* (s^−1^)	*k_cat_*/*K_M_* (s^−1^·mM^−1^)	Reference
WT^ATP^	126.7	0.37	2.94	this study
WT^NR^	85.4	1.86	21.76	this study
G8S^ATP^	42.1	0.66	15.73	this study
G8S^NR^	65.8	2.85	43.31	this study
HNRK	31.00	5.64	0.182	[13]
KPNRK	13.00	3.03	0.244	[13]
KLUNRK	1.98	5.01	2.530	[13]
SPANRK	10.15	3.445	0.353	[13]
BEANRK	8.30	3.38	0.400	[13]

Given variations in assay conditions across studies, the data presented herein should be regarded as a general reference for relative performance rather than as absolute benchmarks.

**Table 2:** Summary of Enzymatic NMN Synthesis Through Different ATP Recycling Systems.

Substrates	Enzymes	ATP Recycling Systems	Enzyme Loading (U/mL)	NMN Production (g/L)	Productivity (g/L/h)	Reference
NR, ATP	*Hi*-NRK^G8S^	ADK	*Hi*-NRK^G8S^: 30 ADK: 10	97.50	32.50	this study
NR, ATP	NRK (*Kl. marxianus*)	ACK + polyP	/	93.50	11.69	[19]
NR, ATP	NRK (*Homo*)	PPK2 + polyP	NRK: 2.5	49.50	24.75	[43]
NR, ATP	NRK^D45T/I88R/E189A^	PPK2 + polyP	Whole-cell	15.16	1.26	[33]
NR, ATP	NRK1 (*S. cerevisiae*)	PPK2 + polyP	NRK1: whole-cell PPK2: 21.9	14.60	1.82	[29]
NAM, ATP, Ribose	NAMPT (*C. pinensis*)	PPK2 + polyP	Whole-cell	48.80	6.10	[44]
NAM, AMP, R5P	NAMPT (*H. ducreyi*)	AMP + RPPK + PPK2 + polyP	NAMPT: 0.5 RPPK: 0.5 PPK2: 0.5	3.00	0.33	[18]
NAM, PRPP	NAMPT^Y13G/Y15S/F76P^ (*C. pinensis*)	PPK + polyP	NAMPT: 1.25	19.94	6.65	[45]
NAM, Ribose	NAMPT^Y15S^ (*C. pinensis*)	PPK + PPase + polyP	NAMPT: 0.5 PRS: 0.5 RK: 0.5 PPK: 0.5 PPase: 0.1	8.10	2.70	[46]

NR: nicotinamide riboside; NRK: nicotinamide riboside kinase; ADK: Adenylate kinase; ACK: acetate kinase; polyP: polyphosphate; PPK: polyphosphate kinase; NAM: Nicotinamide; NAMPT: nicotinamide phosphoribosyltransferase; PPase: pyrophosphatase; R5P: ribose-5-phosphate; RPPK: phosphoribosyl pyrophosphorylase; PRPP: phosphoribosyl pyrophosphate; PRS: Phosphoribosyl Pyrophosphate Synthetase.

## Data Availability

The original contributions presented in this study are included in the article/Supplementary Materials. Further inquiries can be directed to the corresponding author.

## Supplementary Materials

The following supporting information can be downloaded at https://www.mdpi.com/article/10.3390/foods15111838/s1, Table S1: The primers used in this study; Table S2: PCR amplification system; Table S3: PCR amplification program; Figure S1: Ramachandran Plot of the generated structural model of *Hi*-NRK. Figure S2: Sequence alignment of *Hi*-NRK, HNRK, SPANRK, PANRK, KLUNRK and BEANRK; Figure S3: SDS-PAGE plots of *Hi*-NRK with different concentrations of imidazole; Figure S4: Schematic diagram of temperature stability of WT and variants at 40 °C; Figure S5: Kinetic curves of *Hi*-NRK^WT^ and *Hi*-NRK^G8S^ with ATP and NR as substrates. Figure S6: (A) HPLC chromatogram of NR, ATP, NMN, ADP and AMP. (B) HPLC chromatogram of the purified NMN product. Figure S7: (A) Mass spectrum of NMN standard. (B) Mass spectrum of the purified NMN product. Figure S8: The ^1^H NMR data of NMN.

## Abbreviations

The following abbreviations are used in this manuscript:

NMN	nicotinamide mononucleotide
NRK	nicotinamide riboside kinase
NR	nicotinamide riboside
ADK	adenylate kinase
ATP	adenosine triphosphate
AMP	adenosine monophosphate
NAD^+^	nicotinamide adenine dinucleotide
MD	molecular dynamics
MM-PBSA	molecular mechanics Poisson–Boltzmann surface area
DCCM	dynamic cross-correlation matrices
RMSD	root-mean-square deviation
RMSF	root-mean-square fluctuation
NAMPT	nicotinamide phosphoribosyltransferase
PRPP	phosphoribosyl pyrophosphate
NAM	nicotinamide
ACK	acetate kinase
polyP	polyphosphate
PPK	polyphosphate kinase
PPase	pyrophosphatase
R5P	ribose-5-phosphate
RPPK	phosphoribosyl pyrophosphorylase
