# Supplementary material for: Molecular Engineering of Nicotinamide Riboside Kinase and Process Optimization for Efficient Nicotinamide Mononucleotide Production
Source: Foods. 2026 May 22;15(11):1838. doi: 10.3390/foods15111838 (PMC13256911; doi:10.3390/foods15111838)
Supplement: Supplementary file 1 [file foods-15-01838-s001.zip › foods-4288909-supplementary.pdf]

Supplementary Material

Molecular Engineering of Nicotinamide Riboside Kinase and Process Optimization for Efficient Nicotinamide Mononucleotide Production

Dai Ma, Rui Liu, Tong Bao, Jingwen Yang, Hongbin Zhang, Xueqin Hu*

School of Food and Biological Engineering, Hefei University of Technology, Anhui, China 230000

Xueqin Hu*: huxq@hfut.edu.cn ,18855120205

Table S1 The primers used in this study

Primer	Sequence (5'-3')	Primer	Sequence (5'-3')
G8A-F	GCACGTGAATTTCGCCCAGCGCTGCGCATGC	L244R-R	TCTCACGCTTTTGCCGCTGCTTTCACCGCC
G8A-R	GGCGAAATTCACGTGCGGTGGTAAAGCCCATGG	V265R-F	TACGGCCGCGAATTCAGATTCGAGAAGCTG
G8C-F	TGCCGTGAATTTCGCCCAGCGCTGCGCATGC	V265R-R	TCTGAATTCGCGGCCGTATTCCCACGCGCT
G8C-R	GGCGAAATTCACGTGCGGTGGTAAAGCCCATGG	F266A-F	GGCCGCGAATTCGTTGCAGAGAAGCTGGGC
G8D-F	GACCGTGAATTTCGCCCAGCGCTGCGCATGC	F266A-R	TGCAACGAATTCGCGGCCGTATTCCCACGC
G8D-R	GGCGAAATTCACGGTCGGTGGTAAAGCCCATGG	F266C-F	GGCCGCGAATTCGTTTGCGAGAAGCTGGGC
G8E-F	GAACGTGAATTTCGCCCAGCGCTGCGCATGC	F266C-R	GCAAACGAATTCGCGGCCGTATTCCCACGC
G8E-R	GGCGAAATTCACGTTCGGTGGTAAAGCCCATGG	F266D-F	GGCCGCGAATTCGTTGACGAGAAGCTGGGC
G8F-F	TTCCGTGAATTTCGCCCAGCGCTGCGCATGC	F266D-R	GACAACGAATTCGCGGCCGTATTCCCACGC
G8F-R	GGCGAAATTCACGGAAGGTGGTAAAGCCCATGG	F266E-F	GGCCGCGAATTCGTTGAAGAGAAGCTGGGC
G8H-F	CATCGTGAATTTCGCCCAGCGCTGCGCATGC	F266E-R	TTCAACGAATTCGCGGCCGTATTCCCACGC
G8H-R	GGCGAAATTCACGATGGGTGGTAAAGCCCATGG	F266H-F	GGCCGCGAATTCGTTCACGAGAAGCTGGGC
G8I-F	ATACGTGAATTTCGCCCAGCGCTGCGCATGC	F266H-R	GTGAACGAATTCGCGGCCGTATTCCCACGC
G8I-R	GGCGAAATTCACGTATGGTGGTAAAGCCCATGG	F266I-F	GGCCGCGAATTCGTTATAGAGAAGCTGGGC
G8K-F	AAACGTGAATTTCGCCCAGCGCTGCGCATGC	F266I-R	TATAACGAATTCGCGGCCGTATTCCCACGC
G8K-R	GGCGAAATTCACGTTTGGTGGTAAAGCCCATGG	F266K-F	GGCCGCGAATTCGTTAAAGAGAAGCTGGGC
G8L-F	CTACGTGAATTTCGCCCAGCGCTGCGCATGC	F266K-R	TTTAACGAATTCGCGGCCGTATTCCCACGC
G8L-R	GGCGAAATTCACGTAGGGTGGTAAAGCCCATGG	F266L-F	GGCCGCGAATTCGTTCTAGAGAAGCTGGGC
G8M-F	ATGCGTGAATTTCGCCCAGCGCTGCGCATGC	F266L-R	TAGAACGAATTCGCGGCCGTATTCCCACGC
G8M-R	GGCGAAATTCACGCATGGTGGTAAAGCCCATGG	F266M-F	GGCCGCGAATTCGTTATGGAGAAGCTGGGC
G8N-F	AACCGTGAATTTCGCCCAGCGCTGCGCATGC	F266M-R	CATAACGAATTCGCGGCCGTATTCCCACGC
G8N-R	GGCGAAATTCACGGTTGGTGGTAAAGCCCATGG	F266N-F	GGCCGCGAATTCGTTAACGAGAAGCTGGGC
G8P-F	CCACGTGAATTTCGCCCAGCGCTGCGCATGC	F266N-R	GTTAACGAATTCGCGGCCGTATTCCCACGC
G8P-R	GGCGAAATTCACGTGGGGTGGTAAAGCCCATGG	F266P-F	GGCCGCGAATTCGTTCCAGAGAAGCTGGGC
G8Q-F	CAACGTGAATTTCGCCCAGCGCTGCGCATGC	F266P-R	TGGAACGAATTCGCGGCCGTATTCCCACGC
G8Q-R	GGCGAAATTCACGTTGGGTGGTAAAGCCCATGG	F266Q-F	GGCCGCGAATTCGTTCAAGAGAAGCTGGGC
G8R-F	AGACGTGAATTTCGCCCAGCGCTGCGCATGC	F266Q-R	TTGAACGAATTCGCGGCCGTATTCCCACGC

G8R-R	GGCGAAATTCACGTCTGGTGGTAAAGCCCATGG	F266R-F	GGCCGCGAATTCGTTAGAGAGAAGCTGGGC
G8S-F	AGCCGTGAATTTCGCCCAGCGCTGCGCATGC	F266R-R	TCTAACGAATTCGCGGCCGTATTCCCACGC
G8S-R	GGCGAAATTCACGGCTGGTGGTAAAGCCCATGG	F266S-F	GGCCGCGAATTCGTTAGCGAGAAGCTGGGC
G8T-F	ACACGTGAATTTCGCCCAGCGCTGCGCATGC	F266S-R	GCTAACGAATTCGCGGCCGTATTCCCACGC
G8T-R	GGCGAAATTCACGTGTGGTGGTAAAGCCCATGG	F266T-F	GGCCGCGAATTCGTTACAGAGAAGCTGGGC
G8V-F	GTACGTGAATTTCGCCCAGCGCTGCGCATGC	F266T-R	TGTAACGAATTCGCGGCCGTATTCCCACGC
G8V-R	GGCGAAATTCACGTACGGTGGTAAAGCCCATGG	F266V-F	GGCCGCGAATTCGTTGTAGAGAAGCTGGGC
G8W-F	TGGCGTGAATTTCGCCCAGCGCTGCGCATGC	F266V-R	TACAACGAATTCGCGGCCGTATTCCCACGC
G8W-R	GGCGAAATTCACGCCAGGTGGTAAAGCCCATGG	F266W-F	GGCCGCGAATTCGTTTGGGAGAAGCTGGGC
G8Y-F	TACCGTGAATTTCGCCCAGCGCTGCGCATGC	F266W-R	CCAAACGAATTCGCGGCCGTATTCCCACGC
G8Y-R	GGCGAAATTCACGGTAGGTGGTAAAGCCCATGG	F266Y-F	GGCCGCGAATTCGTTTACGAGAAGCTGGGC
F11D-F	ACCACCGGCCGTGAAGACCGCCCAGCGCTG	F266Y-R	GTAAACGAATTCGCGGCCGTATTCCCACGC
F11D-R	GACTTCACGGCCGGTGGTAAAGCCCATGGA	F266G-F	GGCCGCGAATTCGTTGGAGAGAAGCTGGGC
F11Y-F	ACCACCGGCCGTGAATACCGCCCAGCGCTG	F266G-R	TCCAACGAATTCGCGGCCGTATTCCCACGC
F11Y-R	GTATTCACGGCCGGTGGTAAAGCCCATGGA	N272D-F	GAGAAGCTGGGCGGCGACGAACAAGCCATG
N22S-F	ATGCGCGCGAAGTACAGCGCCAAATATCTG	N272D-R	GTCGCCGCCCAGCTTCTCGAAAACGAATTC
N22S-R	GCTGTACTTCGCGCGCATGCGCAGCGCTGG	T311K-F	GACACCGACTTCATCAAAACGCAAGCCTTC
V243T-F	AGCAGCGGCAAAAGCACACTCGTGAACAAA	T311K-R	TTTGATGAAGTCGGTGTCGATGAACGCGAT
V243T-R	TGTGCTTTTGCCGCTGCTTTCACCGCCCAG	D350R-F	AACACCGAGTGGGTGAGAGATGGTCTGCGT
V243K-F	AGCAGCGGCAAAAGCAAACTCGTGAACAAA	D350R-R	TCTCACCCACTCGGTGTTGTTCTTGAGCAG
V243K-R	TTTGCTTTTGCCGCTGCTTTCACCGCCCAG	G357Y-F	GGTCTGCGTAGTCTGTACAGCCAGAAACAG
L244R-F	AGCGGCAAAAGCGTGAGAGTGAACAAACTG	G357Y-R	GTACAGACTACGCAGACCATCATCCACCCA

Table S2 PCR amplification system

Component	50ul system
DNA Template	1ul
Forward primers	1ul
Reverse primers	1ul
5×Fast Buffer	10ul
dNTPs	4ul
dd H_2O	Up to 50ul

Table S3 PCR amplification program

Temperature	Time	
94℃	2 min	
94℃	20 s	25 cycles
55℃	10 s	25 cycles
72℃	3 min	25 cycles
72℃	10 min	

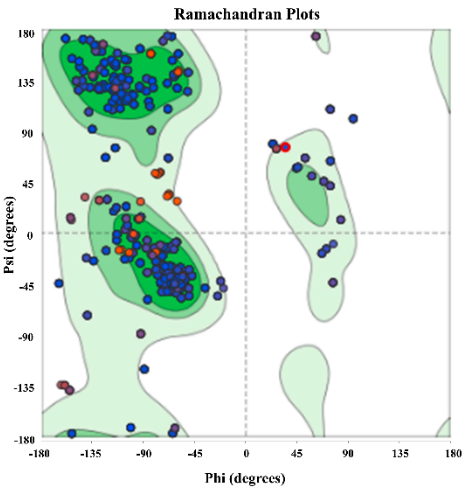

Figure S1: Ramachandran Plot of the generated structural model of *Hi*-NRK.

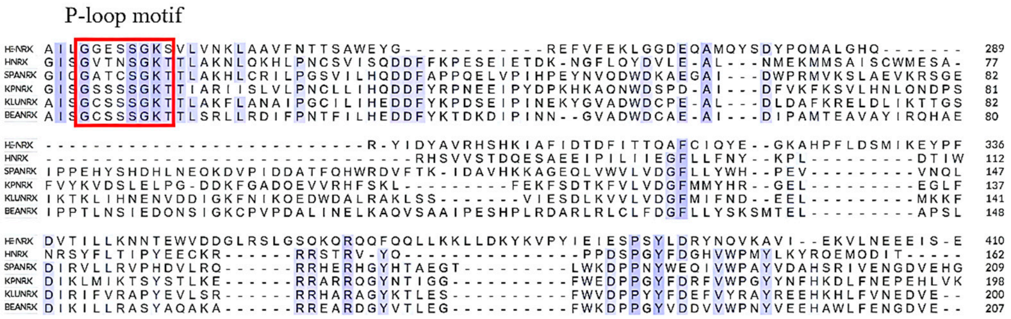

Figure S2: Sequence alignment of *Hi*-NRK, HNRK, SPANRK, PANRK, KLUNRK and BENNRK. Strictly conserved residues are highlighted by a blue background.

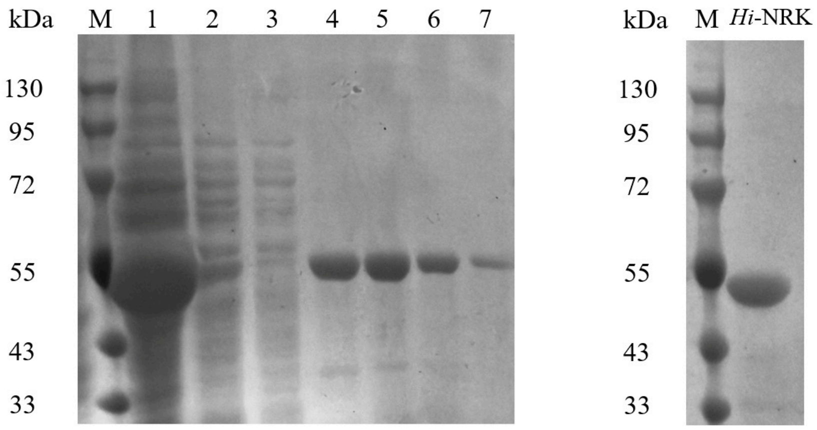

Figure S3: SDS-PAGE plots of *Hi*-NRK with different concentrations of imidazole. From left to right are labelled: M: Marker, 1: crude enzyme solution, 2: crude enzyme effluent, 3: equilibrium buffer effluent, 4-7: 100 mM, 200 mM, 300 mM and 400 mM imidazole eluent, *Hi*-NRK: purified protein.

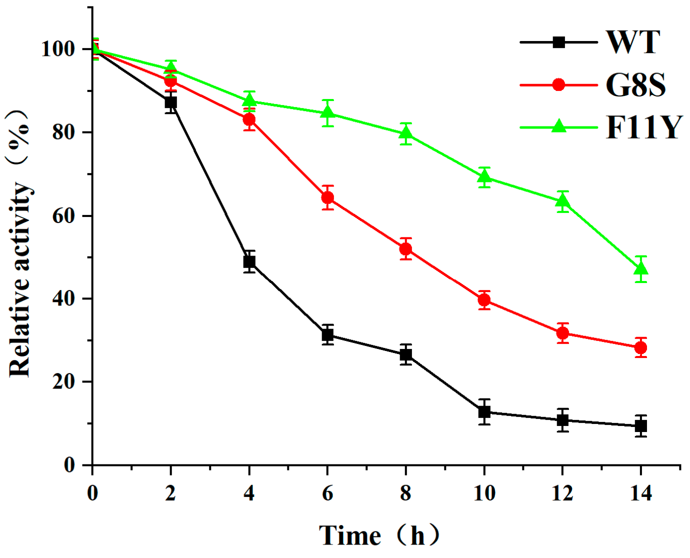

Figure S4: Schematic diagram of temperature stability of WT and variants at 40°C.

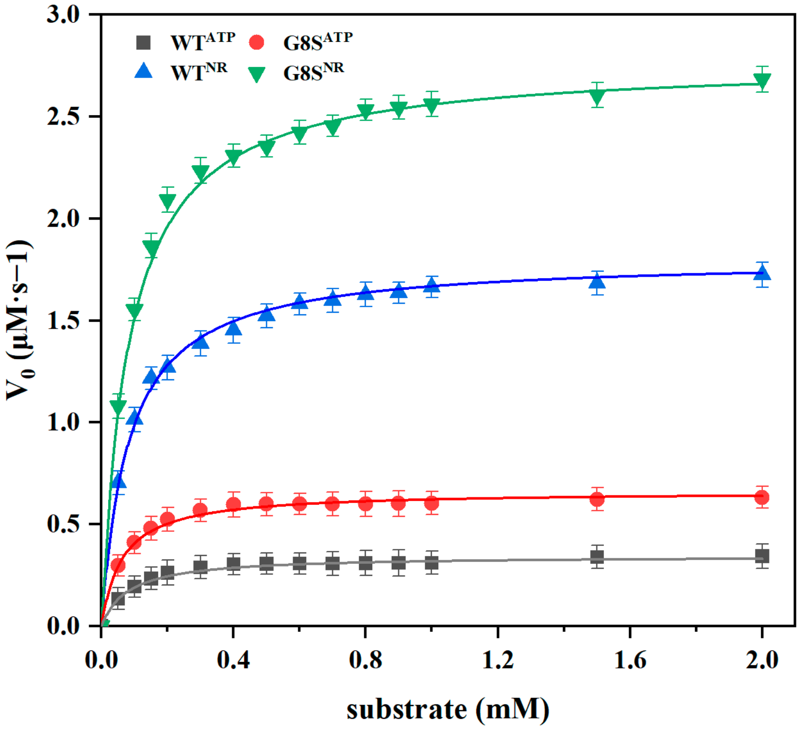

Figure S5: Kinetic curves of *Hi*-NRK^{WT} and *Hi*-NRK^{G8S} with ATP and NR as substrates.

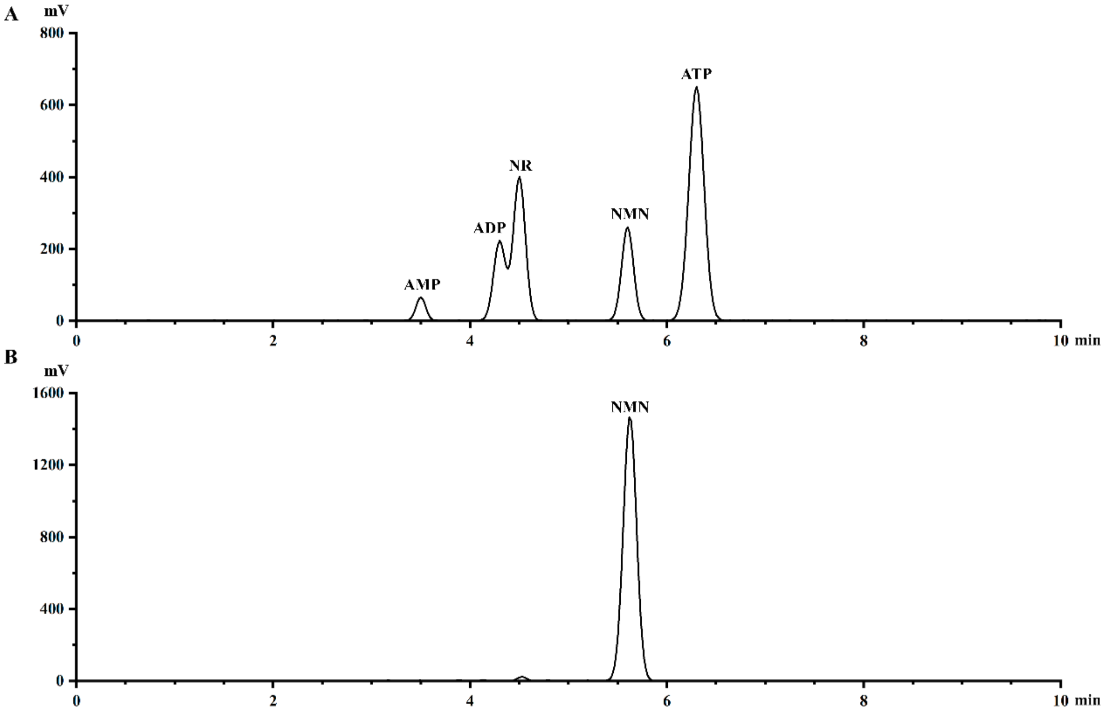

Figure S6: (A) HPLC chromatogram of NR, ATP, NMN, ADP and AMP. (B) HPLC chromatogram of the purified NMN product.

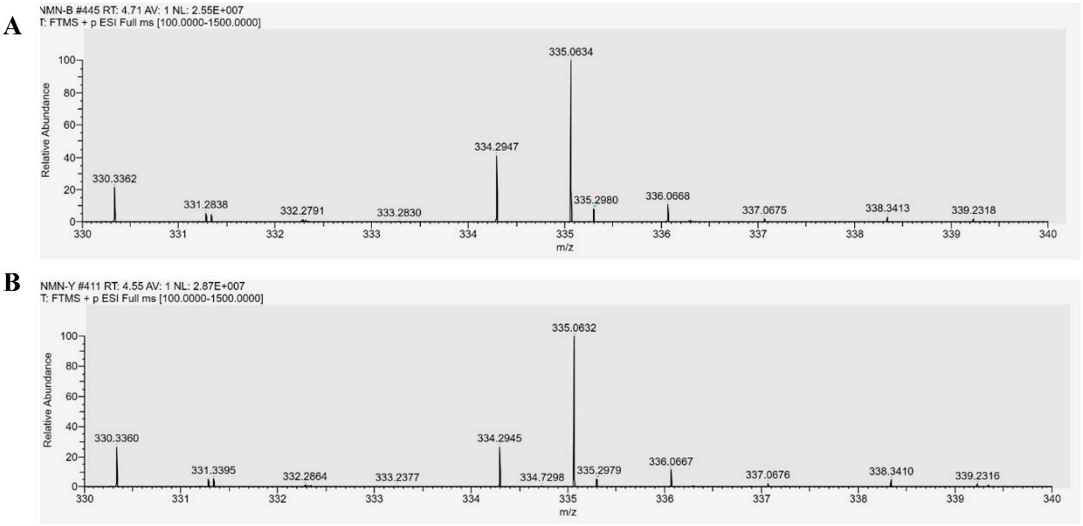

Figure S7: (A) Mass spectrum of NMN standard. (B) Mass spectrum of the purified NMN product.

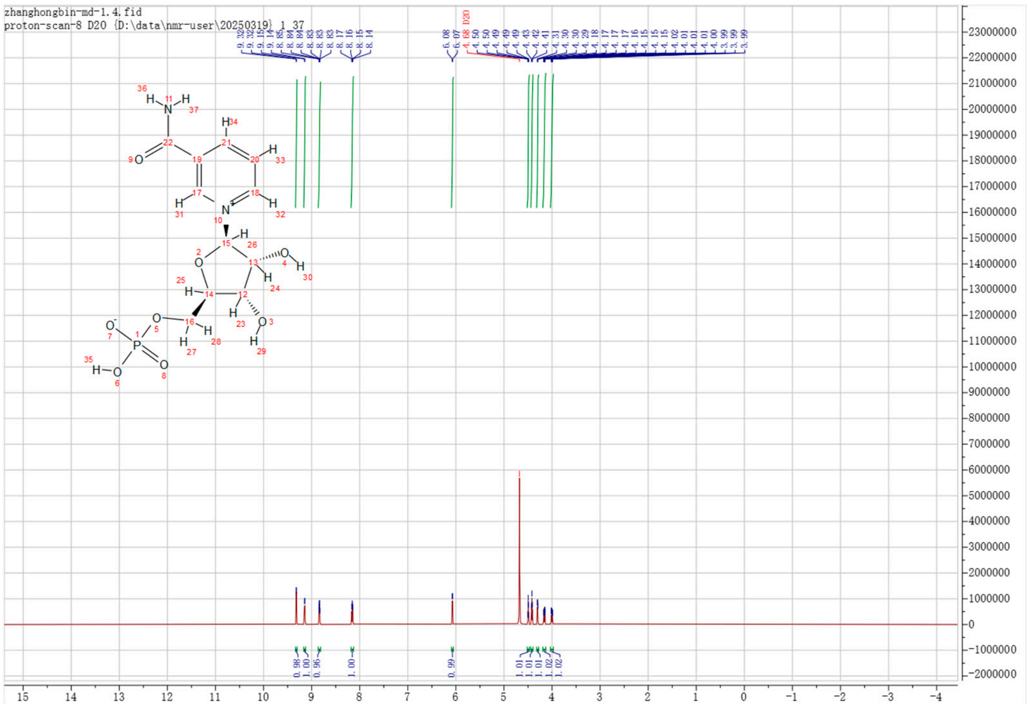

Figure S8: The ^{1}H NMR data of NMN (500 MHz, Deuterium Oxide).δ9.46 (t, J = 1.6 Hz, ^{1}H), 9.29 (dt, J = 6.4, 1.4 Hz, ^{1}H), 8.98 (dt, J =8.1, 1.5 Hz, ^{1}H), 8.30 (dd, J = 8.1, 6.3 Hz, ^{1}H), 6.21 (d, J = 5.5 Hz, ^{1}H), 4.64(p, J = 2.5 Hz, ^{1}H), 4.56 (t, J = 5.2 Hz, ^{1}H), 4.44 (dd, J = 5.0, 2.6 Hz, ^{1}H), 4.31 (ddd, J = 11.9, 4.3, 2.4 Hz, ^{1}H), 4.15 (ddd, J = 12.0, 5.0, 2.2 Hz, ^{1}H).

>*Hi*-NRK

MGFTTGREFHPALRMRAKYNAKYLGTKSEREKYFHLAYNKHTQFLRYQEQIMSKTKEKK
VGVIFGKFYPVHTGHINMIYEAFSKVDELHVIVCSDTVRDLKLFYDSKMKRMPTVQDRLR
WMQQIFKYQKNQIFIHHLVEDGIPSYPNGWQSWSEAVKTLFHEKHFEPSIVFSSEPQDKAP
YEKYLGLEVSLVDPDRTFFNVSATKIRTTPFQYWKFIPKEARPFFAKTVAILGGESSGKSVL
VNKLAAVFNTTSAWEYGREFVFEKLGGDEQAMQYSDYPQMALGHQRYIDYAVRHSHKIA
FIDTDFITTQAFCIQYEGKAHPFLDSMIKEYPFDVTILLKNNTEWVDDGLRSLGSQKQRQQ
FQQLLKKLLDKYKVPYIEIESPSYLDRYNQVKAVIEKVLNEEEISELQNTTFPIKGTSQ

>HNRK

MKTFIIGISGVTNSGKTTLAKNLQKHLPNCSVISQDDFFKPESEIETDKNGFLQYDVLEALN
MEKMMSAISCWMESARHSVVSTDQESAEEIPILIIEGFLLFNYKPLDTIWNRSYFLTIPYEE
CKRRRSTRVYQPPDSPGYFDGHVWPMYLKYRQEMQDITWEVVYLDGTKSEEDLFLQVY
EDLIQELAKQKCLQVTA

>KLUNRK

MTTTKVKLIAISGCSSSGKTTLAKFLANAIPGCILIHEDDFYKPDSEIPINEKYGVADWDCP
EALDLDAFKRELDLIKTTGSIKTKLIHNENVDDIGKFNIKQEDWDALRAKLSSVIESDLKV
VLVDGFMIFNDEELMKKFDIRIFVRAPYEVLSRRRHARAGYKTLESFWVDPPYYFDEFVY
RAYREEHKHLFVNEDVEGSLRSDAGLFELINDDETEITKALNTIADYIVSHLDAN

>SPANRK

MKKRVIFVGIGGATCSGKTTLAKHLCRILPGSVILHQDDFAPPQELVPIHPEYNVQDWDKA
EGAIDWPRMVKSLAEVKRSGEIPPEHYSHDHLNEQKDVPIDDATFQHWRDVFTKIDAVHK
KAGEQLVWVLVDGFLLYWHPEVVNQLDIRVLLRVPHDVLRQRRHERHGYHTAEGTLWK
DPPNYWEQIVWPAYVDAHSRIVENGDVEHGQPNGAVPGLILIDGLEMKMGDMVERVCVR
LAEMVDGEAMRQRDGVDGIVALDHN

>KPNRK

MTQKVVIIGISGSSSSGKTTIARIISLVLPNCLLIHQDDFYRPNEEIPYDPKHKAQNWDSPDA
IDFVKFKSVLHNLQNDPSFVYKVDSLELPGDDKFGADQEVVRHFSKLFEKFSDTKFVLVD
GFMMYHRGELEGLFDIKLMIKTSYSTLKERRARRQGYNTIGGFWEDPPGYFDRFVWPGY
YNFHKDLFNEPEHLVKANGGTLNAFATEQLGIMAFQNDDGGSFKQLQQDVLQSLYDRCIT
LGI

>BEANRK

MGDRKALVVAISGCSSSGKTTLSRLLRDIFPNTFILHEDDFYKTDKDIPINNGVADWDCAE
AIDIPAMTEAVAYIRQHAEIPPTLNSIEDQNSIGKCPVPDALINELKAQVSAAIPESHPLRDA
RLRLCLFDGFLLYSKSMTELAPSLDIKILLRASYAQAKARREARDGYVTLEGFWKDPPGY
VDDVVWPNYVEEHAWLFENGDVEGEYRQEVLQKWGIVVPKGGALDPDMAETLQWMV
DIILTELQRHN
